# Metabolism of Estrogens: Turnover Differs between Platinum-Sensitive and -Resistant High-Grade Serous Ovarian Cancer Cells

**DOI:** 10.3390/cancers12020279

**Published:** 2020-01-23

**Authors:** Stefan Poschner, Judith Wackerlig, Dan Cacsire Castillo-Tong, Andrea Wolf, Isabel von der Decken, Tea Lanišnik Rižner, Renata Pavlič, Anastasia Meshcheryakova, Diana Mechtcheriakova, Monika Fritzer-Szekeres, Theresia Thalhammer, Walter Jäger

**Affiliations:** 1 Division of Clinical Pharmacy and Diagnostics, Department of Pharmaceutical Chemistry, University of Vienna, 1090 Vienna, Austria; stefan.poschner@univie.ac.at; 2 Division of Drug Design and Medicinal Chemistry, Department of Pharmaceutical Chemistry, University of Vienna, 1090 Vienna, Austria; judith.wackerlig@univie.ac.at; 3 Translational Gynecology Group, Department of Obstetrics and Gynecology, Comprehensive Cancer Center, Medical University of Vienna, 1090 Vienna, Austria; dan.cacsire-castillo@meduniwien.ac.at (D.C.C.-T.); andrea.wolf@meduniwien.ac.at (A.W.); isabel.vonderdecken@meduniwien.ac.at (I.v.d.D.); 4 Institute of Biochemistry, Faculty of Medicine, University of Ljubljana, 1000 Ljubljana, Slovenia; tea.lanisnik-rizner@mf.uni-lj.si (T.L.R.); renata.pavlic@mf.uni-lj.si (R.P.); 5 Department of Pathophysiology and Allergy Research, Center for Pathophysiology, Infectiology and Immunology, Medical University of Vienna, 1090 Vienna, Austria; anastasia.meshcheryakova@meduniwien.ac.at (A.M.); diana.mechtcheriakova@meduniwien.ac.at (D.M.); theresia.thalhammer@meduniwien.ac.at (T.T.); 6 Department of Medical and Chemical Laboratory Diagnostics, Medical University of Vienna, 1090 Vienna, Austria; monika.fritzer-szekeres@meduniwien.ac.at; 7 Vienna Metabolomics Center (VIME), University of Vienna, 1090 Vienna, Austria

**Keywords:** high-grade serous ovarian cancer, steroid hormones, metabolomics, LC-HRMS, carboplatin resistance, interleukin-6

## Abstract

High-grade serous ovarian cancer (HGSOC) is currently treated with cytoreductive surgery and platinum-based chemotherapy. The majority of patients show a primary response; however, many rapidly develop drug resistance. Antiestrogens have been studied as low toxic treatment options for HGSOC, with higher response rates in platinum-sensitive cases. Mechanisms for this difference in response remain unknown. Therefore, the present study investigated the impact of platinum resistance on steroid metabolism in six established HGSOC cell lines sensitive and resistant against carboplatin using a high-resolution mass spectrometry assay to simultaneously quantify the ten main steroids of the estrogenic metabolic pathway. An up to 60-fold higher formation of steroid hormones and their sulfated or glucuronidated metabolites was observed in carboplatin-sensitive cells, which was reversible by treatment with interleukin-6 (IL-6). Conversely, treatment of carboplatin-resistant cells expressing high levels of endogenous IL-6 with the monoclonal anti-IL-6R antibody tocilizumab changed their status to “platinum-sensitive”, exhibiting a decreased IC_50_ value for carboplatin, decreased growth, and significantly higher estrogen metabolism. Analysis of these metabolic differences could help to detect platinum resistance in HGSOC patients earlier, thereby allowing more efficient interventions.

## 1. Introduction

Epithelial ovarian cancer (EOC), the most lethal type of gynecological cancer, is the fourth leading cause of cancer-associated mortality among women in the USA and Europe [1,2]. Although the total incidence in 2018 was relatively low with 300,000 new cases worldwide, its fatality rate is high, as the number of deaths was almost 200,000 in the same year [3]. The most frequent form of EOC, accounting for almost 75% of all cases, is high-grade serous ovarian cancer (HGSOC), an aggressive subtype that shows only a 30% to 40% five-year survival rate for all patients [4,5,6].

Cytoreductive debulking surgery and platinum-based chemotherapy is the standard for HGSOC treatment [7,8]. However, although most patients demonstrate a good primary response, the majority (80% to 90%) relapses and develops drug resistance within one year [9]. Novel therapeutic approaches with targeted therapeutics, including poly ADP-ribose)-polymerase 1 (PARP) and vascular endothelial growth factor (VEGF) inhibitors can increase cytotoxic activity and apoptosis in platinum-resistant HGSOC when given in combination with cisplatin or carboplatin [10]. However, not all HGSOC patients are sensitive to PARP and VEGF inhibitors [11]. Moreover, the majority of patients are likely to develop drug resistance against these targeted therapies [12]. Therefore, women diagnosed with recurrent platinum-resistant HGSOC have a poor survival rate, often fewer than 12 months [13]. Improving the therapeutic outcome by preventing drug resistance as long as possible requires the use of alternative treatment strategies. Preclinical studies have shown that estrogens can promote the proliferation of ovarian cancer cells lines and fuel tumor growth in mouse xenograft models [14], which is partly blocked by antiestrogens. Therefore, the use of endocrine disrupting agents in HGSOC could be promising [15]. Indeed, treatment of women with recurrent HGSOC using the antiestrogen tamoxifen or the aromatase inhibitor letrozole resulted in response rates between 10% and 15% and disease stabilization rates of 30% to 40% [16].

Clinical studies have demonstrated that the overall response rate of an endocrine therapy is significantly higher in platinum-sensitive cases as compared with platinum-resistant HGSOC patients (55% vs. 40%) [17], independent of the estrogen receptor alpha (ERα) status of the tumor [18]. Mechanisms for these different response rates of antiestrogens against platinum-sensitive and -resistant HGSOC cells remain unknown. In vitro data reported by Ren et al. [19] indicate altered cellular steroid metabolism, as estrone (E1) is differentially metabolized in normal human ovarian surface epithelium as compared with epithelial ovarian cancer cells SKOV-3 and PEO-1, with higher sulfation rates of E1 and 17β-estradiol (E2) in the noncancerous ovarian surface epithelium cells. Recent studies have also demonstrated that HGSOC cells should be able to inactivate estrogens, as sulfotransferase 1E1 (SULT1E1), a key enzyme responsible for the sulfation of E1, E2, and dehydroepiandrosterone (DHEA), was detected in tumor sections from 137 HGSOC patients by immunohistochemical staining. Notably, multivariate Cox regression analysis revealed that SULT1E1 abundance is a significant predictor for overall survival, as sulfated metabolites exhibit no or only minimal estrogenic activity [20].

How estrogens are metabolized in HGSOC cells and whether platinum resistance affects the formation rates of biotransformation products, particularly that of the most potent estrogen E2, remains unknown. Therefore, the present study simultaneously quantified for the first time the metabolism of the ten major steroids of the estrogenic pathway in HGSOC cell lines sensitive and resistant against carboplatin. Using a validated liquid chromatography high-resolution mass spectrometry (LC-HRMS) assay [21], we were able to selectively determine the levels of precursor steroids, active estrogens, and sulfated or glucuronidated conjugates, which should then be correlated with the platinum sensitivity of the respective cell lines. Furthermore, we screened all six cell lines for endogenous interleukin-6 (IL-6) formation, as IL-6 treatment of A2780 ovarian cancer cells was shown to induce platinum resistance [22]. IL-6 was also described as a marker for platinum resistance in 32 EOC patients [23,24], and elevated levels of IL-6 in the serum and ascites of ovarian cancer patients at the time of diagnosis correlated with a poor initial response to chemotherapy and poor prognosis [25]. Such comprehensive analysis holds promise for a better understanding of drug-resistance mechanisms in HGSOC, which could allow earlier and more efficient interventions for this lethal cancer subtype.

## 2. Results

### 2.1. Characterization of the Investigated HGSOC Cell Lines

**Figure 1:**
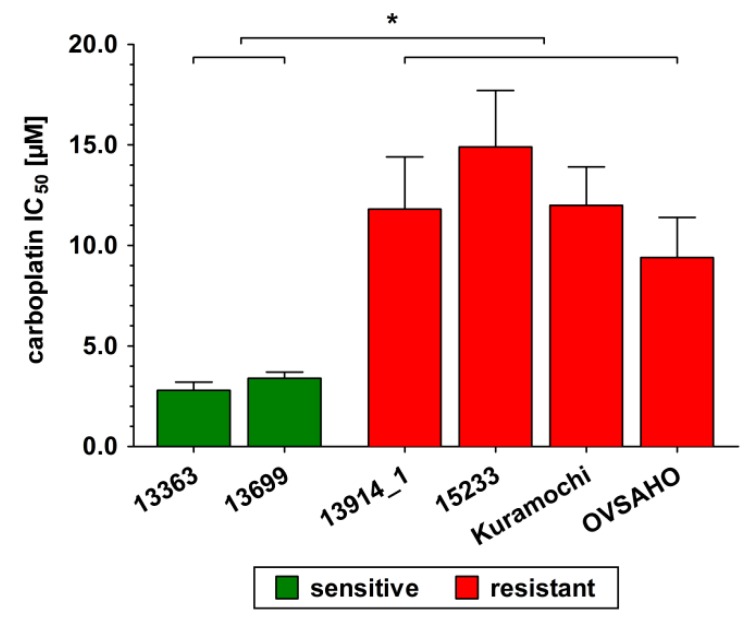
Sensitivity of all investigated high-grade serous ovarian cancer (HGSOC) cell lines in response to carboplatin. Cells were incubated in the presence of increasing carboplatin concentrations (0 to 50 µM) for 72 h and the remaining viable cells were determined using a CASY^®^ TT cell counter. Green color indicates sensitivity and red color indicates resistance against carboplatin to the respective cell line. All data are presented as the means ± SD of three independent experiments. * *p* < 0.05.

As criteria for HGSOC, all six investigated cell lines produce high levels of p53 and PAX8 and carry a mutation in *TP53*. The 13914_1 cell line has an additional mutation in *BRCA1*, the OVSAHO cells in *BRCA2*, and the Kuramochi cells in *BRCA2* and *KRAS*. *EGFR*/*ERBB2* was strongly expressed in all cell lines. A moderate/high expression of *ESR1* and *AR* was only seen in 13699 cells and in Kuramochi cells, whereas a moderate expression of *ESRRG* was found in 13363 and Kuramochi cells. Expression of *ESR2* and *PGR* was low in all six investigated cell lines. All relevant mutations and gene expressions are given in detail in Tables S1 and S2. To classify all cell lines as “platinum-sensitive” or “platinum-resistant”, their respective IC_50_ values against carboplatin were determined over a concentration range of 0–50 µM for 72 h. As shown in Figure 1, 13363 and 13699 cells were highly sensitive to carboplatin with IC_50_ values of 2.8 ± 0.4 and 3.4 ± 0.3 µM, respectively. 13914_1, 15233, Kuramochi, and OVSAHO cells demonstrated three to five times higher IC_50_ values (11.8 ± 2.6, 14.9 ± 2.8, 12.0 ± 1.9, and 9.4 ± 2.0 µM, respectively), and therefore were classified as “platinum-resistant”.

### 2.2. DHEA Metabolism by Platinum-Sensitive and -Resistant HGSOC Cells

**Figure 2:**
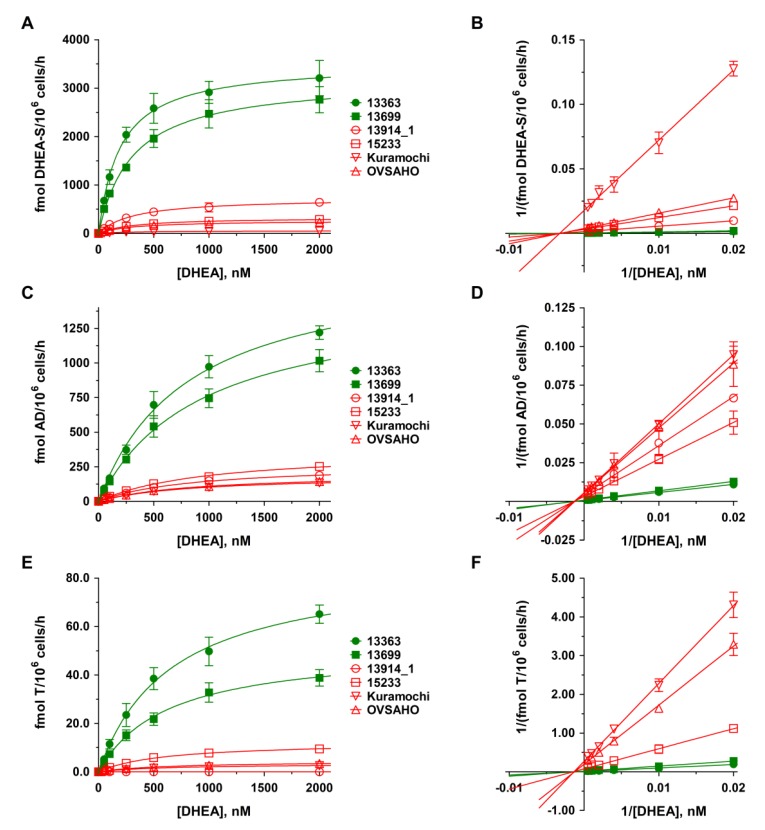
Kinetic profiles of dehydroepiandrosterone (DHEA) metabolite formation in platinum-sensitive and -resistant HGSOC cells. The kinetics of (**A**–**B**) DHEA sulfation, (**C**–**D**) AD formation, and (**E**–**F**) T formation were calculated following the incubation of all HGSOC cell lines with 0 to 2000 nM DHEA as a hormone precursor for 48 h. Data are displayed as Michaelis–Menten and Lineweaver–Burk plots and represent the means ± SD of three independent experiments. Green curves indicate sensitivity and red curves indicate resistance against carboplatin to the investigated HGSOC cell lines. Differences were statistically significant between these two groups (*p* < 0.05).

To investigate the biotransformation of steroids in relation to platinum resistance, all six cell lines were incubated with DHEA (500 nM) and the formation of the nine major human metabolites, namely dehydroepiandrosterone-3-sulfate (DHEA-S); 4-androstene-3,17-dione (AD); testosterone (T); E1, E2, estriol (E3; 16α-hydroxy-17β-estradiol); estrone-3-sulfate (E1-S); 17β-estradiol- 3-sulfate (E2-S); and 17β-estradiol-3-*O*-(β-d-glucuronide) (E2-G) was quantified using a previously validated LC-HRMS assay [21]. Control samples containing dimethylsulfoxide (DMSO) only were also performed to ensure that there was no endogenous steroid formation. After adding 500 nM DHEA, the three biotransformation products DHEA-S, AD, and T could be quantified in addition to parent DHEA in the cellular supernatants (Figure 2). Other biotransformation products could not be detected, indicating no aromatase (CYP19A1) activity. Indeed, *CYP19A1* expression was near the lower limit of detection (LLOQ) in all six cell lines (Section 4.3).

As the levels of metabolites are strongly dependent on incubation time, the number of viable cells and the used steroid precursor concentrations, we decided to show the formation rates (in fmol/10^6^ cells/h) and not absolute concentrations to better allow a comparison between the two carboplatin-sensitive and four carboplatin-resistant HGSOC cell lines. In the platinum-sensitive cell lines 13363 and 13699, sulfation of DHEA to inactive DHEA-S was clearly the favored metabolic pathway, with formation rates of 2583.1 ± 306.9 and 1958.5 ± 184.2 fmol/10^6^ cells/h, respectively. In addition, approximately 20% of DHEA was oxidized to AD via 3β-hydroxysteroid-dehydrogenase (3β-HSD) activity (13363: 697.2 ± 96.5; 13699: 541.9 ± 77.3 fmol/10^6^ cells/h), which was then further converted to T by the action of 17β-hydroxysteroid-dehydrogenase (17β-HSD); however, to a significantly lower extent of only approximately 5% (13363: 38.5 ± 4.5 and 13699: 21.8 ± 2.6 fmol/10^6^ cells/h).

In the platinum-resistant cell lines 13914_1, 15233, Kuramochi, and OVSAHO, the formation rates of DHEA-S, AD, and T were notably lower (maximum 20%) as compared with the platinum-sensitive cells. The formation of DHEA-S was significantly less pronounced (13914_1: 444.2 ± 31.5, 15233: 199.5 ± 9.9, Kuramochi: 32.1 ± 5.3, and OVSAHO: 165.5 ± 15.5 fmol/10^6^ cells/h) and in the same range as the formation of AD (13914_1: 100.2 ± 11.6, 15233: 127.9 ± 13.5, Kuramochi: 72.8.1 ± 5.6, and OVSAHO: 76.6 ± 1.4 fmol/10^6^ cells/h). Formation of T was negligible in 15233 cells (7.7 ± 0.4 fmol/10^6^ cells/h), Kuramochi cells (2.1 ± 0.2 fmol/10^6^ cells/h), and OVSAHO cells (2.6 ± 0.2 fmol/10^6^ cells/h), and undetectable in the 13914_1 cell line.

### 2.3. E1 Metabolism by Platinum-Sensitive and -Resistant HGSOC Cells

To determine estrogen biotransformation, all six cell lines were also incubated with 500 nM E1 as a precursor steroid. Control samples (DMSO) again demonstrated no endogenous estrogen metabolites.

**Figure 3:**
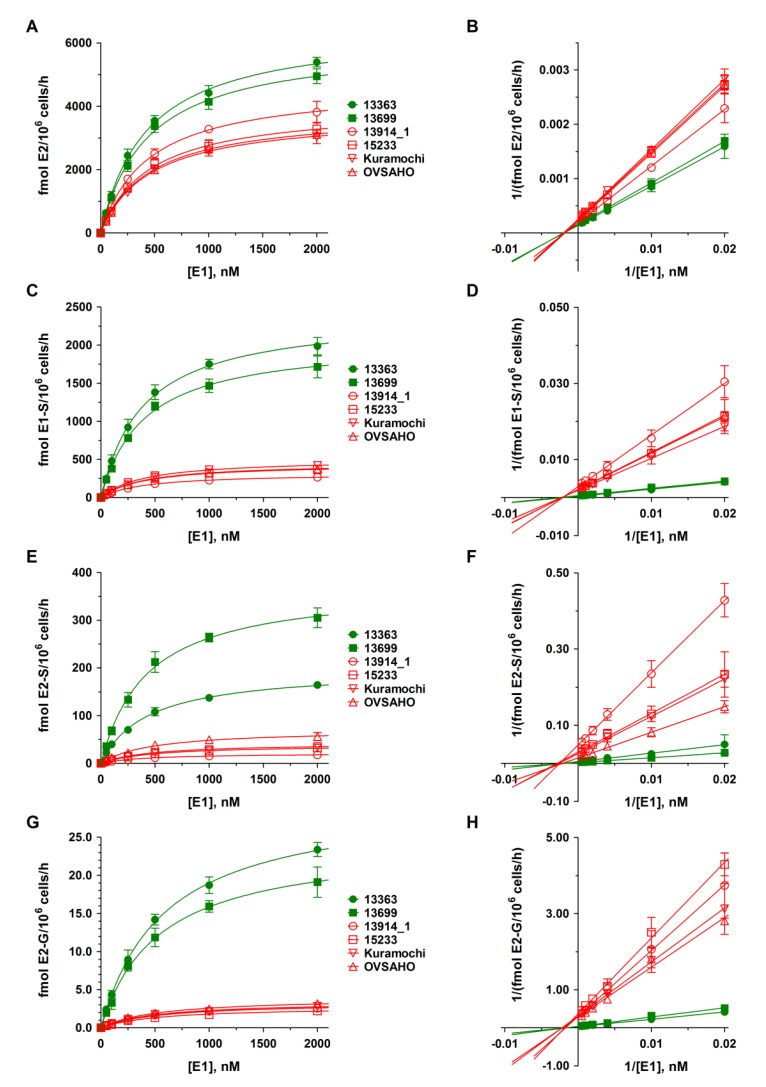
Kinetic profiles of E1 metabolite formation in platinum-sensitive and -resistant HGSOC cells. The kinetics of (**A**–**B**) E2 formation, (**C**–**D**) E1 sulfation, (**E**–**F**) E2 sulfation, and (**G**–**H**) E2 glucuronidation were calculated following the incubation of all HGSOC cell lines with 0 to 2000 nM E1 as a hormone precursor for 48 h. Data are displayed as Michaelis–Menten and Lineweaver–Burk plots and represent the means ± SD of three independent experiments. Green curves indicate sensitivity and red curves indicate resistance against carboplatin to the investigated HGSOC cell lines. Differences were statistically significant between these two groups (*p* < 0.05).

As presented in Figure 3, E2 was the predominant steroid in all HGSOC cell lines, with higher formation rates (3545.6 ± 162.7 and 3374.1 ± 200.2 fmol/10^6^ cells/h) in the platinum-sensitive 13363 and 13699 cells. In the four platinum-resistant cell lines, formation rates were markedly lower (13914_1: 2506.9 ± 149.3, 15233: 2180.8 ± 143.5, Kuramochi: 2030.0 ± 13.3, and OVSAHO: 1997.1 ± 126.5 fmol/10^6^ cells/h). Both E1 and E2 were further conjugated to inactive E1-S and E2-S, and to a minor extent to E2-G, while the CYP3A4-mediated hydroxylation of E2 to E3 could not be quantified in any of the cell lines, due to marginal expression levels of this enzyme (Section 4.3). Again, the formation rates differed markedly between platinum-sensitive and -resistant HGSOC cells. While conjugation of E1 to E1-S amounted to 1381.4 ± 97.2 and 1199.3 ± 53.4 fmol/10^6^ cells/h in the 13363 and 13699 cells, respectively, the E1-S formation rates were up to eight-fold lower in the resistant cell lines (13914_1: 179.4 ± 12.0, 15233: 263.6 ± 16.4, Kuramochi: 286.7 ± 46.9, and OVSAHO: 250.3 ± 19.0 fmol/10^6^ cells/h). Additionally, the formation of E2-S was up to 18-fold higher in the platinum-sensitive HGSOC cells (13363: 108.2 ± 8.7 and 13699: 212.3 ± 21.7 fmol/10^6^ cells/h) as compared with the platinum-resistant HGSOC cells (13914_1: 11.7 ± 1.5, 15233: 20.3 ± 1.4, Kuramochi: 22.8 ± 2.2, and OVSAHO: 38.9 ± 1.9 fmol/10^6^ cells/h). Glucuronidation of E2 to E2-G was only a minor pathway in all investigated cell lines, and was again up to 11-fold lower in platinum-resistant cells (13914_1: 1.7 ± 0.1, 15233: 1.3 ± 0.1, Kuramochi: 1.8 ± 0.2, and OVSAHO: 1.9 ± 0.2 fmol/10^6^ cells/h) as compared with the platinum-sensitive cells (13363: 14.2 ± 0.7 and 13699: 11.9 ± 1.2 fmol/10^6^ cells/h).

### 2.4. Kinetics of DHEA and E1 Metabolism in HGSOC Cells

Kinetic profiles for DHEA and E1 metabolites in all HGSOC cell lines were subsequently evaluated over a DHEA and E1 concentration range of 0 to 2000 nM for 48 h. As demonstrated in Figure 2 and Figure 3, the formation kinetics of all seven quantified metabolites (DHEA-S, AD, T, E2, E1-S, E2-S, and E2-G) best fitted to a hyperbolic Michaelis–Menten model (R^2^: 0.9943 to 0.9997). Notably, Michaelis constants (K_m_ values) for each metabolite were within a similar range in all six tested cell lines, indicating that the affinities of the same enzymes involved in the biotransformation of DHEA and E1 are comparable between carboplatin-sensitive and -resistant HGSOC cells. The maximum reaction velocities (V_max_ values), however, were significantly higher in platinum-sensitive as compared with platinum-resistant cell lines, supporting lower enzymatic activity in the latter ones. All kinetic parameters are presented in detail in Table 1 and Table 2.

### 2.5. Proliferation of Platinum-Sensitive and -Resistant HGSOC Cell Lines

Subsequently, the proliferation rates of the six HGSOC cell lines were evaluated starting from 1.00 × 10^6^ viable cells/well in the absence of any steroid hormone over a time span of 48 h. While the platinum-sensitive cell lines 13363 and 13699 demonstrated a moderate increase in cell numbers to 1.42 ± 0.25 and 1.24 ± 0.23 × 10^6^ viable cells/well, respectively, all platinum-resistant cell lines revealed significantly higher proliferation (13914_1: 1.61 ± 0.39, 15233: 2.07 ± 0.13, Kuramochi: 2.07 ± 0.10, and OVSAHO: 2.00 ± 0.03 × 10^6^ viable cells/well). Addition of DHEA or E1 (0 to 2000 nM) for 48 h did not further stimulate cellular proliferation, indicating that the growth of the investigated cells is independent of stimulation by these steroid precursors (Figure S1).

### 2.6. Effect of IL-6 on Proliferation, Metabolism, and Carboplatin Resistance of Platinum-Sensitive HGSOC Cells

Formation of IL-6 was increased up to 600-fold in the platinum-resistant 13914_1 cell line (302.6 pg/10^6^ cells/h), whereas all other cell lines demonstrated comparably low rates between 0.12 and 2.75 pg/10^6^ cells/h. These findings are also in line with the gene expression data which revealed high *IL-6* expression only in the 13914_1 cell line (Table S2).

**Figure 4:**
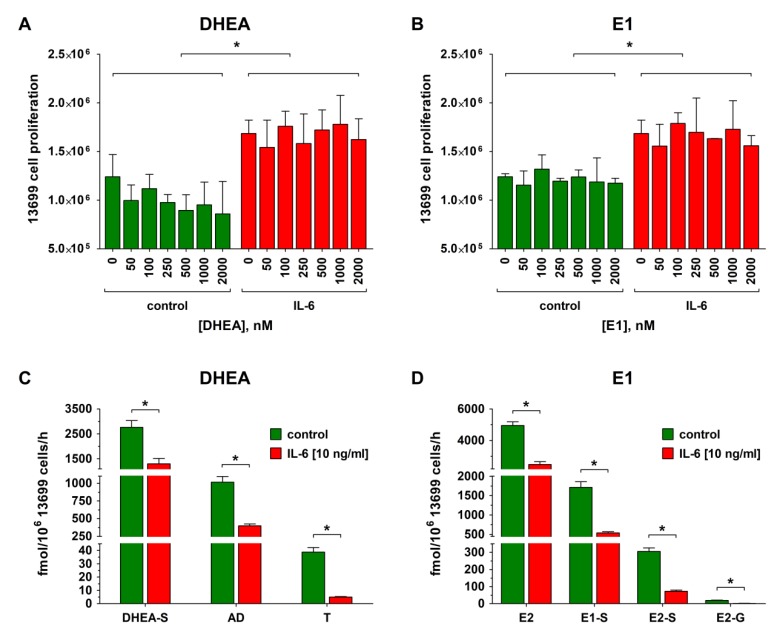
Effect of IL-6 treatment on proliferation and steroid metabolism of 13699 cells. Platinum-sensitive 13699 cells, demonstrating a low endogenous IL-6 formation (2.75 pg/10^6^ cells/h), were incubated with IL-6 (10 ng/mL) for 72 h, followed by incubation with IL-6 in the presence of increasing concentrations (0 to 2000 nM) of (**A**) DHEA and (**B**) E1 for 48 h and the viable cells were counted on a CASY^®^ TT cell counter. Subsequently, the cellular supernatants of the samples containing 2000 nM of the steroid precursor (**C**) DHEA or (**D**) E1 were analyzed for steroid metabolites using liquid chromatography high-resolution mass spectrometry (LC-HRMS). Green indicates sensitivity for carboplatin and red represents carboplatin resistance. All data are presented as the means ± SD of three independent experiments. * *p* < 0.05.

Platinum-sensitive 13699 cells, which exhibit low endogenous IL-6 production and express moderate/high levels of *ESR1* and *AR*, but low levels of *ESR2* and *PGR* (Table S2), were, then, used to investigate whether stimulation of the cells with IL-6 could increase cell proliferation and concomitantly affect estrogen metabolism. As shown in Figure 4A,B, carboplatin-sensitive 13699 cells were treated with IL-6 (10 ng/mL) for 72 h. Afterwards, increasing concentrations of the hormone precursors DHEA or E1 (0 to 2000 nM) were added. IL-6 was further present in the medium and the cellular proliferation was determined after 48 h. Compared with the IL-6 untreated controls, the presence of IL-6 significantly increased cellular growth by 36.3% from 1.24 ± 0.23 to 1.69 ± 0.14 × 10^6^ cells/well. Consistently with the previous experiments (Section 2.5), addition of DHEA or E1 (50 to 2000 nM) to the cells had no further impact on cellular growth.

In contrast to the increased cellular proliferation upon IL-6 treatment, metabolism of DHEA (2000 nM) by 13699 cells to DHEA-S, AD and T was strongly decreased (Figure 4C). While the formation of DHEA-S and AD was reduced by 52.8% and 61.0% (from 2761.2 ± 272.5 to 1302.9 ± 219.1 fmol/10^6^ cells/h and 1016.6 ± 80.3 to 369.7 ± 26.5 fmol/10^6^ cells/h, respectively), the concentration of T was even decreased by 87.1% (from 38.8 ± 3.4 to 5.0 ± 0.3 fmol/10^6^ cells/h). Decreased metabolite formation was also observed upon addition of E1 (2000 nM) in the presence of IL-6. The concentration of unconjugated E2 decreased by 49.9% from 4948.6 ± 232.7 to 2479.5 ± 179.4 fmol/10^6^ cells/h, whereas the decrease of E1-S, E2-S, and E2-G levels was much more pronounced, resulting in a reduction by 68.4%, 76.2%, and 92.2%, respectively (E1-S: 1714.8 ± 143.7 to 541.7 ± 29.9 fmol/10^6^ cells/h, E2-S: 305.0 ± 20.7 to 72.7 ± 6.7 fmol/10^6^ cells/h, and E2-G: 19.1 ± 2.0 to 1.5 ± 0.5 fmol/10^6^ cells/h) (Figure 4D).

Concomitant with these changes in the metabolic activity of 13699 cells, platinum resistance was increased more than three-fold, shifting the IC_50_ against carboplatin from 3.4 ± 0.3 to 11.2 ± 2.4 µM (Figure S2A), indicating that the proinflammatory cytokine IL-6 can convert this cell line from “platinum-sensitive” to “platinum-resistant”.

### 2.7. Effect of Tocilizumab (TCZ) Treatment on Proliferation, Metabolism, and Carboplatin Resistance of Platinum-Resistant HGSOC Cells

**Figure 5:**
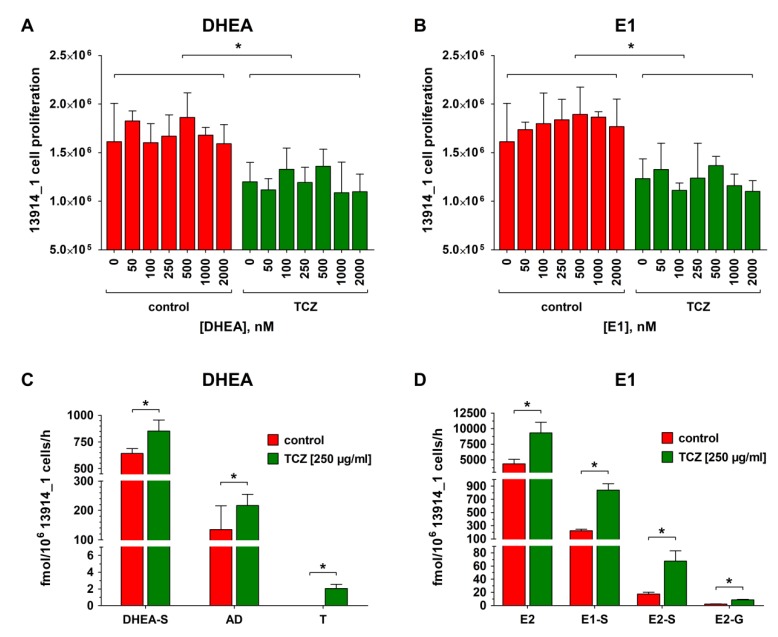
Effect of tocilizumab (TCZ) treatment on the proliferation and steroid metabolism of 13914_1 cell line. Platinum-resistant 13914_1 cells, demonstrating a high endogenous IL-6 formation (302.6 pg/10^6^ cells/h), were incubated with TCZ (250 µg/mL) for 72 h, followed by incubation with TCZ in the presence of increasing concentrations (0 to 2000 nM) of (**A**) DHEA and (**B**) E1 for 48 h and the viable cells were counted on a CASY^®^ TT cell counter. Subsequently, the cellular supernatants of the samples containing 2000 nM of the steroid precursor (**C**) DHEA or (**D**) E1 were analyzed for steroid metabolites using LC-HRMS. Green indicates sensitivity for carboplatin and red represents carboplatin resistance. All data are presented as the means ± SD of three independent experiments. * *p* < 0.05.

To verify that the observed reduced steroid metabolism and increased platinum resistance are indeed related to the action of IL-6, 13914_1 cells (the cell line with the highest endogenous IL-6 formation) were treated with the monoclonal anti-IL-6R antibody TCZ (250 µg/mL) for 72 h before addition of DHEA or E1 (0 to 2000 nM) in the further presence of TCZ for 48 h. As shown in Figure 5A,B, TCZ reduced the proliferation of 13914_1 cells by 25.6% from 1.61 ± 0.39 to 1.20 ± 0.20 × 10^6^ cells/well as compared with the TCZ-untreated controls, while co-incubation with increasing concentrations of DHEA or E1 and TCZ again did not further affect cellular proliferation.

Concomitant with the reduced proliferation of the TCZ-treated 13914_1 cells, the overall metabolic activity significantly increased when DHEA (2000 nM) was added. Formation of DHEA-S and AD increased by 33.0% and 61.0% (DHEA-S: 641.0 ± 47.1 to 852.6 ± 103.7 fmol/10^6^ cells/h and AD: 134.5 ± 80.9 to 216.5 ± 38.7 fmol/10^6^ cells/h). Even T, the formation of which was below the LLOQ in the absence of TCZ, could be now quantified with a formation rate of 2.1 ± 0.5 fmol/10^6^ cells/h (Figure 5C). Also addition of the estrogen precursor E1 (2000 nM) demonstrated a significant increase of E2 by 113.5% (4365.6 ± 718.7 to 9319.8 ± 1703.9 fmol/10^6^ cells/h), which was concomitant with strongly elevated levels of glucuronidated and sulfated metabolites; E2-G increased by 282.0% from 2.2 ± 0.3 to 8.6 ± 0.8 fmol/10^6^ cells/h, whereas E1-S and E2-S were induced by 279.0% and 290.3%, respectively, (221.8 ± 24.2 to 840.4 ± 95.6 fmol/10^6^ cells/h and 17.3 ± 3.0 to 67.7 ± 15.4 fmol/10^6^ cells/h) (Figure 5D).

The TCZ treatment (250 µg/mL) strongly affected also the resistance of 13914_1 cells against carboplatin. While untreated cells demonstrated an IC_50_ value of 11.2 ± 2.4 µM against carboplatin, treatment with the anti-IL-6R antibody decreased this value significantly to 3.4 ± 0.3 µM, therefore, re-establishing the sensitivity for platinum-based chemotherapy in this cell line (Figure S2B).

## 3. Discussion

There is evidence that estrogens play a pivotal role in the progression of ovarian cancer, and that the expression levels of key enzymes vary between benign and cancerous tissues [19]. As differences in the steroid metabolism between platinum-sensitive and -resistant HGSOC cells have not been investigated yet, the present study screened the formation of DHEA and E1 biotransformation products in four recently established and two commercially available HGSOC cell lines as in vitro models for HGSOC.

First, the respective IC_50_ values against carboplatin were determined. Two cell lines were sensitive, whereas the other four cell lines exhibited up to 5.3-fold higher IC_50_ values against carboplatin, and therefore were considered carboplatin-resistant. Notably, 13363 cells, established from a patient prior to chemotherapy, demonstrated sensitivity for carboplatin treatment; while the corresponding 15233 cell line (harvested during chemotherapy) was resistant against carboplatin. These findings are in line with previous data [26,27]. Differences were only found for the OVSAHO cell line, which was considered carboplatin-resistant in the present study but described as cisplatin-sensitive by Haley et al. [27], which is most likely based on different experimental settings, as the MTT assay used by the authors for cell viability measurements is known to generate artifacts causing altered IC_50_ values [28,29].

Following incubation with DHEA, three metabolites, namely DHEA-S, AD, and T, could be quantified in the cellular supernatants. Formation rates of these metabolites were 5- to 60-fold higher in platinum-sensitive cells as compared with the platinum-resistant ones. This is particularly interesting, as the platinum-sensitive 13363 cells were harvested before treatment and the platinum-resistant 15233 cells were harvested during the second cycle of standard platinum-based chemotherapy, thereby, suggesting that the progression of the disease correlates with decreased metabolic activity.

A similar pattern was seen when the cells were incubated with E1. Again, the formation of all metabolites, namely E2, E1-S, E2-S, and E2-G, was significantly higher (up to 1.7-, 7.8-, 17-, and 11-fold, respectively) in carboplatin-sensitive cell lines, with E2, the most potent estrogen, as the main biotransformation product, followed by the sulfated metabolites E1-S and E2-S. E2-G concentrations in the media were low, indicating that sulfation and not glucuronidation is the preferred metabolic pathway in HGSOC cells, which has also been observed in breast cancer [30]. In all six cell lines, hydroxylation of E2 to E3 could not be observed based on low CYP3A4 levels. Conversion of AD to E1 and T to E2 was also not seen in all six cell lines, suggesting no or only very low levels of aromatase (CYP19A1). This is in agreement with the present gene expression analyses and the expression studies by Imai et al. [31], which also detected no aromatase in ovarian cancer cell culture and ovarian carcinoma tissue samples. However, in contrast to the cancer cells, aromatase immunoreactivity was observed in stromal cells adjacent to the tumor [32,33]. Aromatase inhibitors such as letrozole can, therefore, act not on tumor cells directly, but rather indirectly by preventing E2 formation in adjacent cells, thereby, reducing tumor progression and can be a treatment option to increase the progression-free survival of platinum-resistant HGSOC patients via targeting the tumor microenvironment [34].

All four carboplatin-resistant cell lines revealed up to 67% higher proliferation rates as compared with the carboplatin-sensitive cells, independent of the presence of DHEA or E1. Notably, the proliferation rates of the two cell lines derived from the same patient (13363 and 15233 cells) were significantly different (Figure S1). This difference between sensitive and resistant cells was also observed by Xu et al. [35], who reported a higher migration and invasion of platinum-resistant ovarian cancer cells as compared with platinum-sensitive ones, explaining, at least partly, why patients diagnosed with platinum-resistant HGSOC often face faster tumor progression and worse prognosis of the disease.

Recent data showed that autocrine production of the cytokine IL-6 confers cisplatin resistance in ovarian cancer cells [36]. Extracellular IL-6 binds to the cell surface receptor glycoprotein 130 (gp130), thereby activating signaling pathways that promote inflammation, immune reaction, and tumor progression. Elevated serum IL-6 levels in ovarian cancer patients, therefore, correlate with poor prognosis [37]. Most important, elevated IL-6 levels have also been shown to decrease the expression of various estrogen-metabolizing phase I and II enzymes, including members of the cytochrome P450 family (CYPs) and uridine 5’-diphospho-glucuronosyltransferases (UGTs) [38,39,40]. Among other mechanisms, these interactions of IL-6 with E1 and DHEA metabolism is related to the suppression of the nuclear pregnane X receptor (PXR) by IL-6 via JAK/STAT3 signaling, which subsequently leads to a downregulation of genes responsible for estrogen metabolism and transport [41,42,43]. Therefore, it can be hypothesized that increased IL-6 activity in carboplatin-resistant HGSOC cells can contribute to the observed decreased biotransformation of estrogen precursors, increased proliferation rates, and therefore induce platinum resistance.

**Figure 6:**
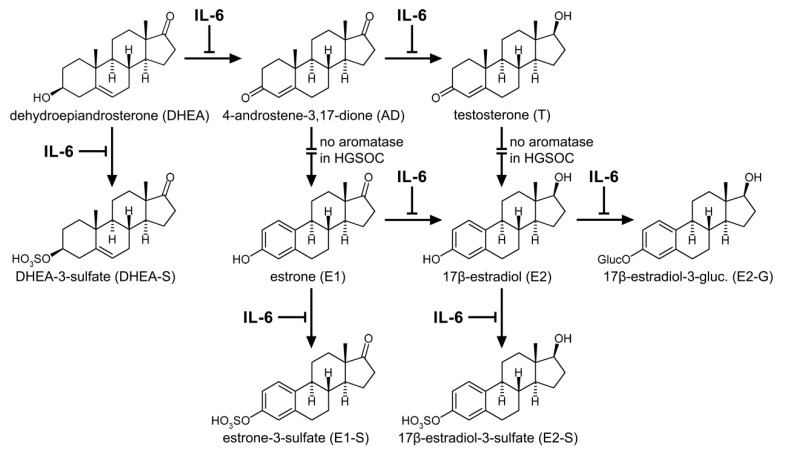
Interaction of IL-6 with the estrogen synthesis and metabolism in HGSOC. IL-6 influences several key steps in the formation of estrogen precursors, active estrogens, and their respective conjugates leading to decreased levels of steroid metabolites. By contrast, treatment with the anti-IL-6 antibody TCZ can antagonize this effect, thereby, stimulating the biotransformation of estrogens. Intersected arrows indicate no expression of aromatase (*CYP19A1*) in HGSOC cells.

To verify this hypothesis, carboplatin-sensitive 13699 cells were treated with recombinant IL-6 for 72 h and, afterwards, their sensitivity for carboplatin, their proliferation rates, and the metabolic activity were again determined. As expected, the presence of IL-6 in the culture medium switched their sensitivity status to “resistant”, with a three-fold higher IC_50_ value, which was concomitant with an increased cellular proliferation and decreased DHEA and E1 metabolism (Figure 6). Consequently, IL-6 can act as a resistance marker for some but not all HGSOC cases. Conversely, treatment of the carboplatin-resistant cell line 13914_1, which expresses high endogenous IL-6 levels, with the IL-6R specific monoclonal antibody TCZ changed the cell status to “sensitive”, demonstrating a decreased IC_50_ value for carboplatin, decreased cellular growth, and significantly higher DHEA and E1 metabolism. These findings are in line with previous data, which have also shown that treatment of EOC cells with TCZ inhibited cellular proliferation, whereas a combination of TCZ with carboplatin further synergistically reduced cell growth [44]. These effects were also observed in paclitaxel-resistant SKOV-3 and CAOV-3 ovarian cancer cells, where the anti-IL-6 antibody siltuximab increased paclitaxel sensitivity, leading to lower cell viability and decreased IC_50_ values [45].

Although TCZ stimulated the conjugation, and therefore the inactivation of estrogens in carboplatin-resistant 13914_1 cells, the formation of E2 via 17β-HSDs was strongly increased and resulted in higher unconjugated E2 concentrations as compared with untreated cells. This elevation of active estrogens upon anti-IL-6R treatment can contribute to disease progression and explain, at least partly, the lack of efficacy of antibody monotherapy in HGSOC [46]. Therapeutic combination of TCZ with standard chemotherapy (carboplatin as a single drug or in combination with paclitaxel [47]) could be a promising treatment strategy to re-establish platinum sensitivity in HGSOC patients. As only patients with high endogenous plasma levels of IL-6 and IL-6R expression could benefit from this therapy, platinum-resistant HGSOC cases have to be screened for IL-6 levels before treatment with a recombinant monoclonal anti-IL-6R antibody.

The altered expression of the genes *ESR1* and *AR* encoding ERα and AR, whose abundances are inhomogeneous in the investigated HGSOC model cell lines, also influence the action of estrogens and, consequently, proliferation of platinum-resistant HGSOC cells. Whereas 13699 and Kuramochi cells express moderate/high levels of *ESR1* and *AR*, but poor levels of *ESR2* and *PGR*, the other investigated cell lines have low or undetectable levels of all steroid hormone receptors (Table S2). Despite these differences in receptor status, incubation of all six HGSOC cell lines with DHEA and E1 did not increase cellular proliferation, thereby, defining these cell lines as hormone independent.

The lack of additional proliferation in the presence of hormone precursors is most likely a consequence of the fact that all cell lines already reached their respective maximum proliferation capacity from the stimulatory effect of numerous other factors. Mutations in *TP53* will fuel tumor cell growth by preventing cell cycle arrest and apoptosis [43], and the moderate/high expression levels of *EGFR* and *ERBB2* in all six cell lines (Table S2) could also contribute to an uncontrolled cellular growth [44].

Our data showed that estrogen metabolism did not correlate with *ESR1*, *ESR2,* and *ESRRG* expression. This is supported by a previous paper by Andersen et al. [48], showing that approximately 80% of HGSOC tumor samples express *ESR1*; however, the response to an antiestrogenic therapy in patients is rather poor. This group also reported that the ERα status in HGSOC cells is not a sufficient tool to predict the response to an antiestrogenic therapy. Other proteins, e.g., IGFBP3, could also be important for the response.

All six cell lines also carry distinct mutations in *TP53* in the DNA binding domain or oligomerization domain (Table S1), leading to a truncated protein. The lack of additional proliferation in the presence of hormone precursors is most likely a consequence of the fact that all cell lines already reached their respective maximum proliferation capacity from the stimulatory effect of various cyclines (e.g., *D1, E1, A1,* and *B)* overexpressed in these cell lines. Genes such as *CDKN1A BAX* or *TIGAR*, controlling apoptosis and cell cycle arrest, are furthermore downregulated [26,49]. These data indicate that the loss of wild type p53 function is the major driving force of tumor cell progression [50]. Additionally, the moderate/high expression levels of *EGFR* and *ERBB2* in all six cell lines (Table S2) could also contribute to an uncontrolled cellular growth [51].

Although our data indicate that estrogen metabolism can differ between platinum-sensitive and -resistant HGSOC cells, clinical data are highly warranted to verify this observation in platinum-resistant cancer patients. We are well aware that several other mechanisms for platinum resistance are known that could be used as clinical markers, including an alteration in cellular accumulation or detoxification of platinum drugs. A decreased expression of the membrane copper transporter CTR1 or the organic cation transporter OCT2, as well as a high expression of the copper-exporting P-type ATPases, ATP7A and ATP7B, or the ATP-binding cassette multidrug transporter, MRP2, could lead to decreased intracellular levels of platinum drugs, thereby causing resistance. Furthermore, high expression levels of glutathione S-transferase, a detoxifying enzyme responsible for the formation of platinum-glutathione conjugates, would also facilitate resistance [52]. A combination of these already identified markers with differences in estrogen metabolism could allow a better prediction of platinum resistance in patients.

## 4. Materials and Methods

### 4.1. Reagents

AD, DHEA, dehydroepiandrosterone-2,2,3,4,4,6-d_6_ (DHEA-d_6_), DHEA-S (sodium salt), dehydroepiandrosterone-3-sulfate-2,2,3,4,4,6-d_6_ (DHEA-S-d_6_ sodium salt), E1, E1-S (sodium salt), E2, E2-G (sodium salt), E3, T, acetic acid, acetonitrile, ammonium acetate, carboplatin, DMSO, and human IL-6 (HumanKine^®^, expressed in HEK 293 cells, suitable for cell culture) were obtained from Merck KGaA (Darmstadt, Germany). All solvents and additives were purchased with HPLC/MS grade purity. 4-Androstene-3,17-dione-2,2,4,6,6,16,16-d_7_ (AD-d_7_), 17β-estradiol-2,4,16,16-d_4_ (E2-d_4_), 17β-estradiol-16,16,17-d_3_-3-*O*-(β-d-glucuronide) (E2-G-d_3_ sodium salt), 17β-estradiol-2,4,16,16-d_4_- 3-sulfate (E2-S-d_4_ sodium salt), estriol-2,4,17-d_3_ (E3-d_3_), estrone-2,4,16,16-d_4_ (E1-d_4_), estrone-2,4,16,16-d_4_-3-sulfate (E1-S-d_4_ sodium salt), and testosterone-2,2,4,6,6-d_5_ (T-d_5_) were obtained from C/D/N-Isotopes Inc. (Pointe-Claire, Quebec, Canada). E2-S (sodium salt) was purchased from Steraloids Inc. (Newport, RI, USA). The anti-IL-6R antibody TCZ (RoActemra^®^) was purchased from Roche Austria GmbH (Vienna, Austria). Water for all experiments was purified using an arium^®^ pro ultrapure water system (Sartorius AG, Göttingen, Germany). If not stated otherwise, all standards were dissolved in DMSO to their final concentration and stored at −80 °C until further usage. All deuterated standards were then mixed to obtain the final internal standard master mix composition. Dulbecco’s modified Eagle medium F-12 (DMEM/F-12), Dulbecco’s phosphate buffered saline (DPBS), fetal bovine serum (FBS), PenStrep^®^, and TrypLe^®^ solutions were purchased from Invitrogen; Thermo Fisher Scientific, Inc. (Waltham, MA, USA). HyClone^®^ heat-inactivated charcoal/dextran treated FBS was obtained from THP Medical Products (Vienna, Austria).

### 4.2. Cell Lines

13363, 13699, 13914_1, and 15233 HGSOC cancer cells, characterized and authenticated via short tandem repeats (STR) profiling as described previously [26], were kindly provided by the Translational Gynecology Group at the Medical University of Vienna. The four cell lines were established from the ascites of three grade 3 HGSOC patients. The 13363 and 15233 cells were harvested from the same patient (age 33 and FIGO: IV); the first ones at the time of diagnosis and the latter ones under the treatment with carboplatin/paclitaxel. The 13699 and 13914_1 cells originated from two patients (both FIGO: IIIC), aged 53 and 66, respectively. Kuramochi (RRID:CVCL_1345) and OVSAHO (RRID:CVCL_3114) cell lines were originally established from undifferentiated ovarian adenocarcinoma [53] and serous papillary ovarian adenocarcinoma [54], respectively, and were described as the best commercial in vitro models for HGSOC [55]. Both cell lines were obtained from the JCRB Cell Bank (Osaka, Japan) which certified the authenticity of their STR profiles. The *P53* and *PAX8* gene expression confirmed that all cell lines were high grade. Cells were routinely cultivated in phenol red-free DMEM/F-12 containing 10% FBS and 1% PenStrep^®^ solution at 37 °C (95% humidity and 5% CO_2_) and the experiments were performed during the exponential growth phases of the cells.

### 4.3. Gene Expression Analyses and Identification of Gene Mutations

Expression of selected genes (*CYP3A4*, *CYP19A1*, *TP53*, *PAX8*, *AR*, *ESR1*, *ESR2*, *PGR*, *IL6*, *EGFR, ERBB2,* and *ESRRG*) in the HGSOC cell lines 13363, 13699, 13914_1, and 15233 was analyzed by next-generation sequencing, as described previously [49]. Expression data for these selected genes in Kuramochi and OVSAHO cells were taken from the GENEVESTIGATOR platform [56]. The *TP53* mutation was determined by a modified p53 functional yeast assay and Sanger sequencing. In addition, ddPCR systems for each unique *TP53* mutation were established to determine the percentage of the *TP53* mutant cells in cell culture. The *BRCA1*, *BRCA2,* and *KRAS* mutations were determined by Sanger sequencing [49]. Data for Kuramochi and OVSAHO cells were obtained from the Cancer Cell Line Encyclopedia (CCLE) [57].

### 4.4. Carboplatin Resistance

To elucidate the sensitivity of all investigated cell lines to carboplatin treatment, cells were seeded in triplicate in 6-well plates at a concentration of 1.00 × 10^6^ cells/well and allowed to attach overnight. Cells were washed with DPBS and incubated with phenol red-free DMEM/F-12 supplemented with 10% FBS and 1% PenStrep^®^ solution at 37 °C (95% humidity and 5% CO_2_) containing 0 to 50 µM carboplatin. Carboplatin was dissolved in sterile-filtered water according to Hall et al. [58] (final concentration 0.1%) before addition to the culture media, as other solvents, such as DMSO, inactivate platinum complexes. After 72 h, the supernatant media were discarded, and the cell layers were washed with DPBS and detached using 400 µL TrypLe^®^ solution. Immediately afterwards, cell suspensions were analyzed for the number of viable cells using a CASY^®^ TT cell counting system (OLS OMNI Life Science, Bremen, Germany), described as an improved method for cell viability measurements at least for platinum complexes, as the MTT assay might generate artifacts leading to altered IC_50_ values [28,29].

### 4.5. Metabolism of Steroid Hormones by Platinum-Sensitive and -Resistant HGSOC Cells

For the metabolomic analyses, cells were cultivated and seeded in 6-well plates, as described in Section 4.4. Prior to the incubation with either DHEA or E1 (0 to 2000 nM) as hormone precursors (dissolved in sterile-filtered DMSO, final concentration 0.05%), the cell layers were washed twice with DPBS, and phenol red-free DMEM/F-12, containing only 10% heat-inactivated charcoal-stripped fetal bovine serum and 1% PenStrep^®^ solution was added to exclude any effects of hormones and growth factors from standard FBS. After 48 h, which was determined in preliminary experiments as the most suitable time point (Poschner, S.; Jäger, W. University of Vienna, Vienna, Austria. Unpublished work, 2019.), the supernatant cell media were collected and stored at −80 °C until further analysis. The remaining cells were detached by adding 400 µL TrypLe^®^ solution and subsequently counted.

Then, 2000 µL media aliquots, mixed with 20 µL deuterated internal standard solution were put onto Oasis HLB 1 cc solid phase extraction cartridges (30 mg; Waters Corporation, Milford, MA, USA), as described previously [21]. Briefly, after preconditioning the cartridges twice with 1.0 mL acetonitrile and three times with 1.0 mL ammonium acetate buffer (10 mM and pH = 5.0), the samples were loaded onto the columns and washed with 1.0 mL ammonium acetate buffer (10 mM and pH = 5.0) and twice with 1.0 mL acetonitrile/ammonium acetate buffer (10 mM and pH = 5.0) 10:90 (*v*/*v*). Analyte elution was, then, achieved by two washes with 650 µl acetonitrile/ammonium acetate buffer (10 mM and pH = 5.0) 95:5 (*v*/*v*), and the samples were left to evaporate until dry. The dried residues were reconstituted in 270 µL acetonitrile/ammonium acetate buffer (10 mM and pH = 5.0, 25:75) (*v*/*v*) and stored until further LC-HRMS analysis at −80 °C.

### 4.6. LC-HRMS Assay for Steroid Quantification

To quantify the ten most prevalent estrogen precursors, active estrogens and their metabolites (DHEA, DHEA-S, AD, T, E1, E2, E3, E1-S, E2-S, and E2-G) in the media samples, a previously established and validated LC-HRMS was used [21]. Chromatographic separation was achieved on an UltiMate 3000 RSLC-series system (Dionex/Thermo Fisher Scientific, Inc.) coupled to a maXis HD ESI-Qq-TOF mass spectrometer (Bruker Daltonics, Bremen, Germany), which was equipped with a Phenomenex Luna^®^ 3 µm C18(2) 100 Å LC column (250 × 4.6 mm I.D.; Phenomenex Inc., Torrance, CA, USA) and a Hypersil^®^ BDS-C18 guard column (5 µm, 10 × 4.6 mm I.D.; Thermo Fisher Scientific, Inc.). Then, 100 µL of the reconstituted media samples were injected onto the column. Chromatographic separation was performed at 43 °C using a continuous gradient mixed from aqueous ammonium acetate buffer (10 mM and pH = 5.0) as mobile phase A and acetonitrile as mobile phase B at a flow rate of 1.0 mL/min. Mobile phase B linearly increased from 25% at 0 min to 56.3% at 19 min, further increased to 90% at 19.5 min and was kept constant until 24.0 min. The percentage of acetonitrile was, then, decreased within 0.5 min to 25% in order to equilibrate the column for 6 min before application of the next sample. The injection volume for each sample was set to 100 µL.

The ESI ion source settings were identical for both modes, except for the polarity: Capillary voltage, ± 4.5 kV; nebulizer, 1.0 bar N_2_; dry gas flow, 8.0 L/min N_2_; and dry temperature, 200 °C. Values for the ion optics and the quadrupole and collision cell parameters were as follows: Funnel RF, 400 Vpp; multipole RF, 300 Vpp; quadrupole ion energy, 8.0 eV; collision RF, 1100 Vpp; collision energy, 10.0 eV; transfer time, 38 ms; and prepulse storage, 18 ms. Full scan mass spectra in the range of m/z 150–500 were recorded in both negative and positive ion mode. The LLOQs for all analytes (signal to noise ratio ≥9) were calculated to be as follows: AD, 74.9 pg/mL; DHEA, 1904.0 pg/mL; DHEA-S, 8.0 pg/mL; E1, 19.0 pg/mL; E1-S, 4.0 pg/mL; E2, 140.9 pg/mL; E2-G, 12.0 pg/mL; E2-S, 3.4 pg/mL; E3, 28.4 pg/mL; and T, 54.1 pg/mL. Quality control samples (containing each analyte at a concentration of 6-, 60- or 600-fold of the respective LLOQ) were performed with each batch.

### 4.7. IL-6 Determination in the Cellular Supernatants

In order to quantify the endogenous IL-6 formation, all HGSOC cell lines were cultivated in T-75 tissue culture flasks (75 cm^2^; BD-Falcon^®^, Thermo Fisher Scientific, Inc.) until confluence. Subsequently, the cell layers were washed twice with 6.0 mL DPBS, and 8.0 mL phenol red-free DMEM/F-12, containing 10% heat-inactivated charcoal-stripped FBS and 1% PenStrep^®^ solution, were added to exclude any external interference with the assay. After incubation for 24 h, the supernatants were collected and stored at −80 °C until further analysis. The remaining cell layers were detached using 2.0 mL TrypLe^®^ solution and counted to obtain the number of viable cells in each flask. The contents of IL-6 in the supernatant media were, then, determined using a commercial cobas^®^ Elecsys IL-6 kit (Roche Diagnostics GmbH, Mannheim, Germany) according to the manufacturer’s instructions.

### 4.8. Impact of IL-6 and TCZ on Metabolism and Progression of HGSOC Cells

To investigate the impact of the proinflammatory cytokine IL-6 on the platinum resistance and estrogen metabolism of HGSOC cells, carboplatin-sensitive 13699 HGSOC cells, which exhibited low endogenous IL-6 formation, were treated with 10 ng/mL IL-6 for 72 h in phenol red-free DMEM/F-12, supplemented with only 10% heat-inactivated charcoal-stripped fetal bovine serum and 1% PenStrep^®^ solution. Then, cells were seeded in 6-well plates in the presence of IL-6 (10 ng/mL), and their sensitivity for carboplatin (0 to 50 µM) was assessed again as described in Section 4.4. Furthermore, IL-6-treated cells were also incubated in the presence of DHEA or E1 (2000 nM) for 48 h (as described in Section 4.5) and the levels of steroid metabolites in the cell supernatants were quantified using the same LC-HRMS assay as mentioned in Section 4.6. The same experimental protocol was used with 250 µg/mL monoclonal anti-IL-6R antibody TCZ in the platinum-resistant 13914_1 cell line, which was the cell line with the highest endogenous IL-6 formation, to investigate whether TCZ can reverse the proinflammatory effects of IL-6 in HGSOC cells.

### 4.9. Data Analysis and Statistics

The acquired LC-HRMS data were analyzed using the Compass DataAnalysis 4.2 and QuantAnalysis 2.2 software packages (Bruker Daltonics). For all analytes and internal standards, extracted ion chromatograms were calculated and the respective peak areas were determined. The ratios of the peak areas of each analyte/internal standard pair were subsequently used for quantification. The kinetic analyses of steroid metabolism in all HGSOC cell lines were then performed using GraphPad Prism 6.0 software (GraphPad Software, Inc., La Jolla, CA, USA) and best followed the Michaelis–Menten model:

V = V_max_ × [S]/(K_m_ + [S]), (1)

where V is the rate of the reaction, V_max_ is the maximum reaction velocity, [S] is the initial substrate concentration, and K_m_ is the Michaelis constant. The same software package was also used for all other calculations and statistical analyses. All experiments were conducted with three independent experiments; and the data were reported as the mean ± standard deviation (SD) of all analyzed samples. One-way ANOVA combined with Tukey’s post-hoc test was used to determine differences between treatment groups and controls, with a statistical significance level of *p* < 0.05.

## 5. Conclusions

Resistance against platinum-based drugs is a main obstacle in the therapy of HGSOC patients. Novel markers can allow earlier and more efficient interventions for this lethal cancer subtype. In the present study, we demonstrated that steroid metabolism significantly differs between carboplatin-sensitive and -resistant HGSOC cells. Further experiments also revealed that treatment of carboplatin-sensitive cells with IL-6 decreased platinum-sensitivity concomitant with a decreased metabolic activity but increased proliferation. Treatment of carboplatin-resistant cells expressing high levels of IL-6 with the anti-IL-6R antibody TCZ also changed their resistance status back to sensitive, now showing increased estrogen metabolism and decreased IC_50_ values against carboplatin. Further studies using tumor specimens from HGSOC patients are warranted to establish estrogen metabolism as a marker for platinum resistance in the clinics.

## Figures and Tables

**Table 1:** Kinetic parameters of DHEA metabolism by the investigated HGSOC cells. K_m_ and V_max_ values were calculated using GraphPad Prism 6.0 software following the incubation of the cell lines with increasing concentrations of DHEA (0 to 2000 nM) as a hormone precursor for 48 h. All data are presented as the means ± SD of three independent experiments. Values in bold and marked with an asterisk (*) are significantly different as compared with both carboplatin-sensitive cell lines 13363 and 13699 (*p* < 0.05). n.c., not calculable.

Cell Line	K_m_ [nM]			V_max_ [fmol/10^6^ Cells/h]		
	DHEA-S	AD	T	DHEA-S	AD	T
Carboplatin-sensitive						
13363	193.9 ± 9.3	812.7 ± 79.7	657.7 ± 52.4	3523.7 ± 47.8	1736.6 ± 75.9	85.6 ± 2.8
13699	308.7 ± 19.2	929.4 ± 68.8	623.5 ± 50.2	3192.4 ± 64.7	1478.8 ± 51.1	51.4 ± 1.7
Carboplatin-resistant						
13914_1	318.7 ± 18.6	854.1 ± 39.9	n.c.	729.0 ± 13.9 *	269.8 ± 5.7 *	0.0 *
15233	314.1 ± 11.1	980.4 ± 88.6	592.0 ± 33.0	328.5 ± 3.8 *	369.7 ± 15.9 *	12.3 ± 0.3 *
Kuramochi	318.5 ± 36.2	841.7 ± 43.4	621.5 ± 45.4	56.9 ± 2.1 *	189.0 ± 4.4*	3.4 ± 0.1 *
OVSAHO	310.8 ± 20.7	871.4 ± 36.7	631.4 ± 30.8	264.8 ± 5.7 *	204.9 ± 3.9 *	4.4 ± 0.1 *

**Table 2:** Kinetic parameters of E1 metabolism by the investigated HGSOC cells. K_m_ and V_max_ values were calculated using GraphPad Prism 6.0 software following the incubation of the cell lines with increasing concentrations of E1 (0–2000 nM) as a hormone precursor for 48 h. All data are presented as the means ± SD of three independent experiments. Values in bold and marked with an asterisk (*) are significantly different as compared with both carboplatin-sensitive cell lines 13363 and 13699 (*p* < 0.05).

K_m_ [nM]				
Cell line	E2	E1-S	E2-S	E2-G
Carboplatin-sensitive				
13363	430.4 ± 22.9	390.5 ± 18.4	426.8 ± 22.1	580.1 ± 20.0
13699	434.8 ± 27.9	399.3 ± 25.5	422.5 ± 29.8	532.9 ± 34.3
Carboplatin-resistant				
13914_1	440.8 ± 13.2	398.0 ± 9.1	418.0 ± 16.8	535.3 ± 39.6
15233	451.4 ± 22.8	407.3 ± 29.0	458.8 ± 38.7	537.0 ± 44.3
Kuramochi	454.0 ± 23.4	402.8 ± 14.1	452.0 ± 23.7	521.2 ± 52.6
OVSAHO	450.0 ± 19.8	403.3 ±18.3	464.6 ± 54.5	534.4 ± 48.5
**V_max_ (fmol/10^6^ Cells/h)**				
**Cell line**	**E2**	**E1-S**	**E2-S**	**E2-G**
Carboplatin-sensitive				
13363	6492.9 ± 124.7	2404.8 ± 39.7	198.3 ± 3.7	30.0 ± 0.4
13699	6028.7 ± 140.4	2067.9 ± 46.6	373.7 ± 9.5	24.3 ± 0.6
Carboplatin-resistant				
13914_1	4687.8 ± 51.2 *	318.9 ± 2.6 *	21.5 ± 0.3 *	3.3 ± 0.1 *
15233	4017.7 ± 74.5 *	455.7 ± 11.5 *	38.8 ± 1.2 *	2.8 ± 0.1 *
Kuramochi	3750.0 ± 71.0 *	508.4 ± 6.3 *	42.7 ± 0.8 *	3.5 ± 0.1 *
OVSAHO	3833.8 ± 61.9 *	443.9 ± 7.1 *	70.8 ± 3.1 *	3.9 ± 0.1 *

## Supplementary Materials

The following are available online at https://www.mdpi.com/2072-6694/12/2/279/s1, Figure S1: Proliferation of HGSOC cell lines in the presence of the hormone precursors DHEA and E1, Figure S2: IC_50_ values of HGSOC cell lines in response to carboplatin in the presence and absence of IL-6 or TCZ, Table S1: Mutations of the investigated HGSOC cell lines, and Table S2: Expression of selected genes in the investigated HGSOC cell lines.

## Abbreviations

17β-HSD	17β-hydroxysteroid-dehydrogenase
3β-HSD	3β-hydroxysteroid-dehydrogenase
AD	4-androstene-3,17-dione
CYP	cytochrome P450
DHEA	dehydroepiandrosterone
DHEA-S	dehydroepiandrosterone-3-sulfate
DMEM/F-12	Dulbecco’s modified Eagle medium F-12
DPBS	Dulbecco’s phosphate buffered saline
E1	estrone
E1-S	estrone-3-sulfate
E2	17β-estradiol
E2-G	17β-estradiol-3-*O*-(β-d-glucuronide)
E2-S	17β-estradiol-3-sulfate
E3	estriol
EOC	epithelial ovarian cancer
ERα	estrogen receptor alpha
*ESR1*	estrogen receptor alpha gene
*ESR2*	estrogen receptor beta gene
*ESRRG*	estrogen-related receptor gamma gene
FBS	fetal bovine serum
HGSOC	high grade serous ovarian cancer
IL-6	interleukin-6
IL-6R	interleukin-6 receptor
K_m_	Michaelis constant
LC-HRMS	liquid chromatography high-resolution mass spectrometry
LLOQ	lower limit of quantification
PARP	poly(ADP-ribose)-polymerase 1
PXR	pregnane X receptor
STR	short tandem repeats
SULT	sulfotransferase
T	testosterone
TCZ	tocilizumab
UGT	uridine 5’-diphospho-glucuronosyltransferase
VEGF	vascular endothelial growth factor
V_max_	maximum reaction velocity
