# Supplementary material for: Metabolism of Estrogens: Turnover Differs between Platinum-Sensitive and -Resistant High-Grade Serous Ovarian Cancer Cells
Source: Cancers (Basel). 2020 Jan 23;12(2):279. doi: 10.3390/cancers12020279 (PMC7072378; doi:10.3390/cancers12020279)
Supplement: Supplementary file 1 [file cancers-12-00279-s001.pdf]

Supplementary Materials

Metabolism of Estrogens: Turnover Differs Between Platinum-Sensitive and -Resistant High-Grade Serous Ovarian Cancer Cells

Stefan Poschner, Judith Wackerlig, Dan Cacsire Castillo-Tong, Andrea Wolf, Isabel von der Decken, Tea Lanišnik Rižner, Renata Pavlič, Anastasia Meshcheryakova, Diana Mechtcheriakova, Monika Fritzer-Szekeres, Theresia Thalhammer and Walter Jäger

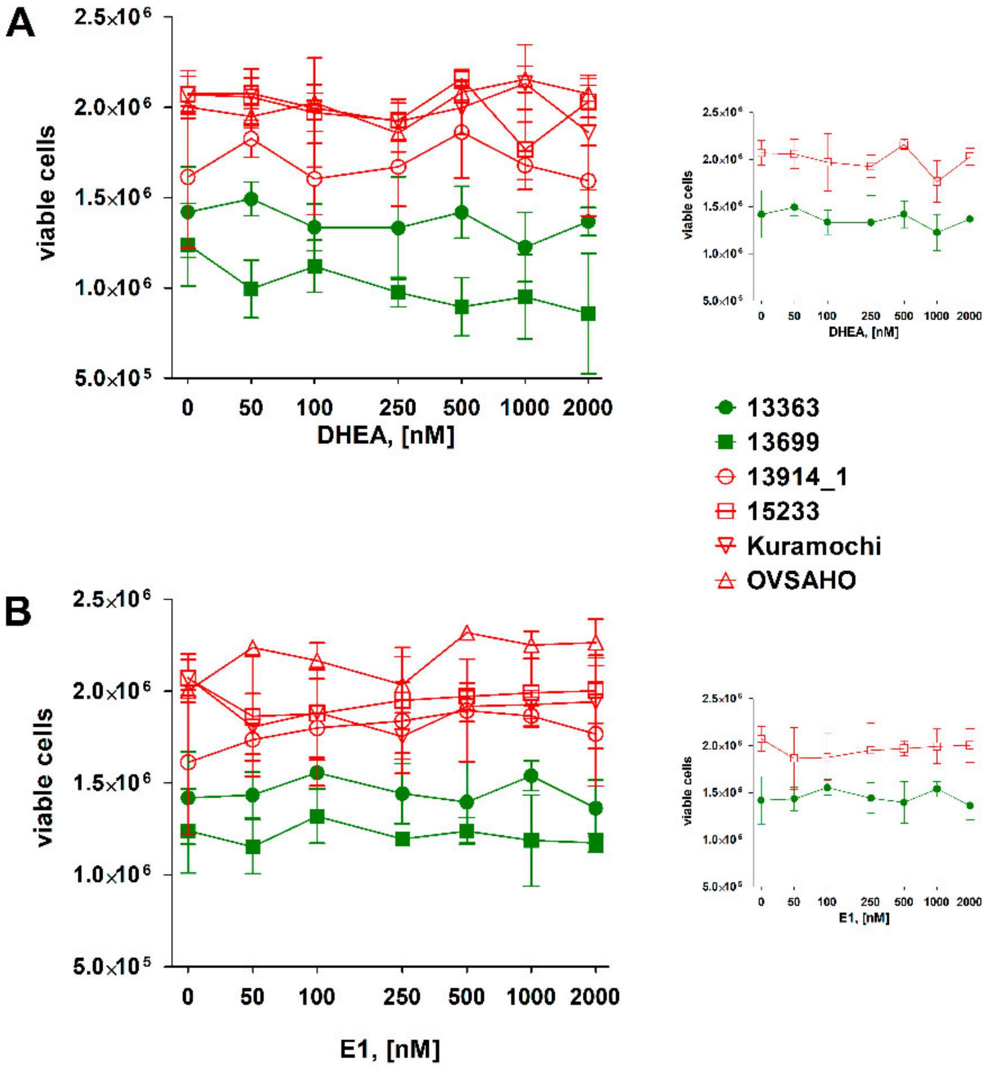

Figure S1. Proliferation of HGSOC cell lines in the presence of the hormone precursors DHEA and E1. Cells were incubated with increasing concentrations (0-2000 nM) of (**A**) DHEA or (**B**) E1 for 48 h. All data are presented as the means ± SD of three independent experiments. Green lines indicate sensitivity and red lines indicate resistance against carboplatin to the investigated HGSOC cell lines. Inserts highlight the different proliferation rates between 13363 and 15233 cells, which were derived from the same patient.

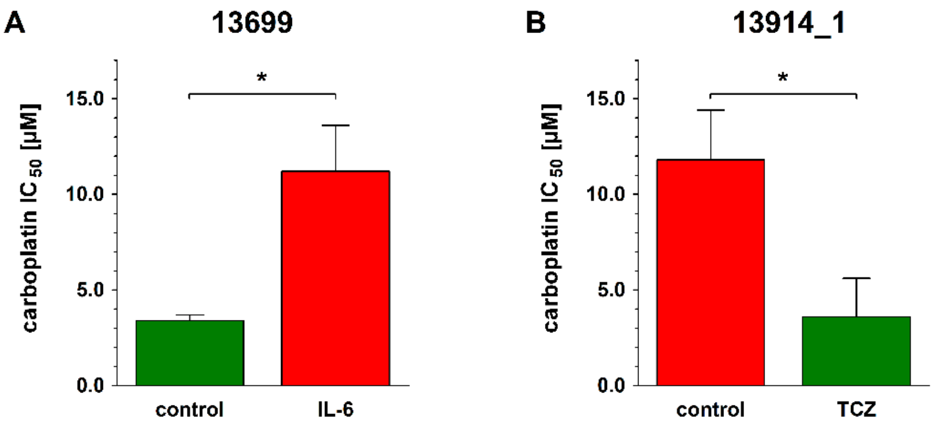

Figure S2. IC_{50} values of HGSOC cell lines in response to carboplatin in the presence and absence of IL-6 or TCZ. (**A**) 13699 cells were treated with IL-6 (10 ng/ml) for 72 h and subsequently exposed to a concentration range of 0-50 µM carboplatin while still exposed to IL-6 for further 72 h, and the remaining viable cells were determined using a CASY® TT cell counter. (**B**) 13914_1 cells were treated with TCZ (250 µg/ml) for 72 h and subsequently exposed to a concentration range of 0-50 µM carboplatin while still exposed to TCZ for further 72 h. Control samples were performed using the same protocol containing vehicle only. The IC_{50} values were then calculated using GraphPad Prism 6.0 software and are reported as the means ± SD of three independent experiments. Green indicates sensitivity for carboplatin, while red represents carboplatin-resistance. *$p < 0.05$.

Table S1. Mutations of the investigated HGSOC cell lines. Mutations of *TP53*, *BRCA1*, *BRCA2* and *KRAS* are given in detail for all six cell lines. Data were collected as stated in section 4.3. W.T., wild-type.

Cell line	*TP53*	*BRCA1*	*BRCA2*	*KRAS*
		Carboplatin-sensitive		
13363	g.13187_13189 del3 (Thr170del)	W.T.	W.T.	W.T.
13699	g.17575 delC (Arg333Valfs*12)	W.T.	W.T.	W.T.
		Carboplatin-resistant		
13914_1	g.17597_17606 del (Met340Serfs*2)	c.3481_3491del (Glu1161Phefs*3)	W.T.	W.T.
15233	g.13187_13189 del3 (Thr170del)	W.T.	W.T.	W.T.
Kuramochi	c.841 C→A (Asp281Tyr)	W.T.	c.6952 C→T (Arg2318Ter)	Ampli17
OVSAHO	c.1024 C→T (Arg342Ter)	W.T.	Del17	W.T.

Table S2. Expression of selected genes in the investigated HGSOC cell lines. Gene expression for 13363, 13699, 13914_1 and 15233 HGSOC cell lines was performed using Sanger sequencing. Data are expressed as read-counts × 10^{-3} [48]. Data for Kuramochi and OVSAHO cells were extracted from GENEVESTIGATOR on the basis of mRNASeq data sets.

Gene	Carboplatin-sensitive		Carboplatin-resistant			
	13363	13699	13914_1	15233	Kuramochi	OVSAHO
TP53	2.87	1.43	2.85	2.71	7.30	3.58
PAX8	3.89	3.95	4.19	3.20	8.02	6.62
ESR1	0.01	1.58	0.16	0.00	3.44	0.18
ESR2	0.02	0.02	0.00	0.00	0.20	0.18
ESRRG	1.48	0.16	0.85	0.21	1.49	0.78
AR	0.00	1.28	0.24	0.00	1.34	0.14
PGR	0.03	0.00	0.00	0.01	0.02	0.00
IL6	0.01	0.18	3.41	0.01	0.65	0.56
EGFR	4.19	5.12	11.24	3.71	2.49	2.29
ERBB2	6.81	6.65	2.86	4.39	6.14	5.04

© 2020 by the authors. Licensee MDPI, Basel, Switzerland. This article is an open access article distributed under the terms and conditions of the Creative Commons Attribution (CC BY) license (http://creativecommons.org/licenses/by/4.0/).
